# Incidence and predictors of attrition among human immunodeficiency virus infected children on antiretroviral therapy in Amhara comprehensive specialized hospitals, Northwest Ethiopia, 2022: a retrospective cohort study

**DOI:** 10.1038/s41598-024-54229-z

**Published:** 2024-02-22

**Authors:** Alemu Birara Zemariam, Gebremeskel Kibret Abebe, Addis Wondmagegn Alamaw

**Affiliations:** 1 Department of Pediatrics and Child Health Nursing, School of Nursing, College of Medicine and Health Sciences, Woldia University, Woldia, Ethiopia; 2 Department of Emergency and Critical Care Nursing, School of Nursing, College of Medicine and Health Sciences, Woldia University, Woldia, Ethiopia

**Keywords:** Antiretroviral therapy, Attrition, Children, Ethiopia, Incidence, Diseases, Health care, Medical research

## Abstract

Attrition rate is higher in developing nations and it leftovers a major obstacle to enhance the benefits of therapy and achieve the 90–90–90 plan targets. Despite this fact, data on the incidence and its predictors of attrition among human immune deficiency virus infected children on antiretroviral therapy are limited in developing countries including Ethiopia especially after the test and treat strategy implemented. This study aimed to assess the incidence and predictors of attrition among human immune deficiency virus infected children on antiretroviral therapy in Amhara Comprehensive Specialized Hospitals, Northwest Ethiopia. A retrospective follow-up study was conducted among 359 children on ART from June 14, 2014, to June 14, 2022. Study participants were selected using simple random sampling method and the data were collected using Kobo Toolbox software and analysis was done by STATA version 14. Both bi-variable and multivariable Cox regression models were fitted to ascertain predictors. Lastly, an AHR with a 95% CI was computed and variables with a p-value of < 0.05 were took an account statistically key predictors of attrition. The overall incidence of attrition rate was 9.8 (95% CI 7.9, 11.9) per 100 PYO. Children having baseline hemoglobin < 10 mg/dl (AHR 3.94; 95% CI 2.32, 6.7), suboptimal adherence (AHR 1.96; 95% CI 1.23, 3.13), baseline opportunistic infection (AHR 1.8; 95% CI 1.17, 2.96), and children who had experienced drug side effects (AHR 8.3; 95% CI 4.93, 13.84) were established to be a significant predictors of attrition. The attrition rate was relatively high. Decreased hemoglobin, suboptimal adherence, presence of drug side effects and baseline opportunistic infection were predictors of attrition. Therefore, it is crucial to detect and give special emphasis to those identified predictors promptly.

## Introduction

Acquired immune deficiency syndrome (AIDS) is an important worldwide public health issues. From a total of 1.7 million children aged under 15, who are affected by human immunodeficiency virus (HIV) worldwide, almost 90% of them were resides in Sub-Saharan Africa^1^. Attrition in the context refers to the disruption or discontinuation of antiretroviral therapy (ART) care. It encompasses various outcomes such as patients being lost to follow-up, experiencing mortality, or discontinuing ART^2^.

The primary actions required to enhance the gains of antiretroviral therapy (ART) and meet the 90–90–90 target plan are early detection, linkage, and minimizing the attrition rate by enhancing the retention rate of children on ART care^3^. Nonetheless, attrition in ART programs continues to be a major concern particularly in resource-constrained nations^4^. Attrition can causes surge the risk of disease transmission, progression, and resistance to numerous ART drug regimens which ultimately resulting in morbidity and mortality^5^.

Children may discontinue receiving ART care for various reasons. These reasons include having limited socioeconomic status, lacking sufficient education to understand the importance of lifelong care, living far from the ART facility without access to transportation, encountering negative attitudes from healthcare providers, experiencing long waiting times, facing drug side effects, dealing with delayed defaulter-tracing systems, struggling with suboptimal adherence to treatment, and lacking social support due to stigma and issues related to disclosure^2,6–8^.

Several studies conducted across countries revealed that there was a high rate of attrition among children on ART care. Such as a retrospective studies conducted in India and Ethiopia showed that a cumulative incidence rate of attrition among children on ART was found to be 24.9% and 20.6% respectively^9,10^. Moreover, a study conducted on the pediatric West African Database revealed that the incidence rate of attrition was 35.8/100 child-years^11^. Being younger, having severe immunosuppression, living in rural areas, having a baseline Hgb < 10 g/dl, being underweight, and having advanced WHO clinical stages were found to be some of the factors associated with attrition among children on ART^7,11^.

Several interventions have been implemented so far by different organizations to reduce the incidence of attrition. These are warranting continual drug supplies, took account of simple, less costly, non-toxic ART regimens and decentralization of ART care in all health facility. In addition, the Ministry of Health of Ethiopia has been developed a strategic plan to improve adherence of patients and minimizing the attrition rate by increasing the retention rate of children on ART care^12^. Though these intervention was implemented so far, the global and national incidence of attrition still high and there were limited evidence about it in resource limited settings in particular after the test and treat strategies implemented. So, this study aimed to figure out this issue.

The quick expansion of ART is one of the most extraordinary attainments in public health history, however attrition remains a critical defy to the quality of ART care and the achievement of ART program. In Ethiopia, a very few studies were conducted to provide an important insight into the incidence and predictors of attrition. However, these studies were conducted in a single-center institution, which precludes the external validity of the study. Besides, the previous few studies were done before the test and treat strategies implemented in Ethiopia despite these strategy showed plentiful positive outcomes compared to the previous pre-strategy era^13^ and its association with attrition has not been fully investigated and understood globally, particularly in resource limited countries like Ethiopia. Hence, it is required to provide updated information on the incidence and predictors of attrition particularly after the test and treat strategy implemented. Therefore, this study aimed to assess the incidence of attrition after the “test and treat era” and its predictors among children on ART in Amhara Comprehensive Specialized Hospital, Northwest Ethiopia. By doing so, this study helps to propose appropriate interventions to reduce the incidence rate, maximize retention, enhance the adherence, and improve the clinical outcome of children on ART care. Besides, the study will help to add the scientific knowledge on this thematic area for future researcher, clinicians, and other stakeholders.

## Methods

### Study design, period and setting

A multicenter retrospective follow-up study design was conducted from June 14, 2014, to June 14, 2022. The study was done in selected Amhara region comprehensive specialized hospitals. These include Debretabor, University of Gondar, Felege Hiwot, and Debre Markos comprehensive specialized hospital. They are located 592 km, 644 km, 492 km, and 298 km away from the capital city of Ethiopia (Addis Ababa), respectively. The hospitals provided services for more than 2.7 million, 7 million, 5 million, and 3.5 million people in the catchment area of their respective orders^14^. Apart from other services, pediatric ART services have been provided in all hospitals since 2005. From June 14, 2014, to June 14, 2022, a total of 684 HIV-infected children were newly initiated ART^15,16^.

### Population of the study

All HIV-infected children whose age less than 15 years initiated ART at Amhara region comprehensive specialized hospitals were considered as the target population and all HIV-infected children less than 15 years of age who started ART from June 14, 2014, to June 14, 2022, in selected Amhara region comprehensive specialized hospitals were considered as the study population.

### Eligibility criteria

All HIV-infected children < 15 years of age who initiated ART from June 14, 2014, to June 14, 2022, in Amhara region comprehensive specialized hospitals were included, whereas HIV-infected children on ART with unrecorded values of the outcome variable (i.e., the status of attrition) and incomplete charts were excluded.

### Sample size determination

For the first objective, a single population formula was employed to calculate the sample size considering the following statistical assumption: P = cumulative incidence of attrition among children on ART was 30.5%^17^. Z α/2 = the corresponding Z score of 95% CI, d = margin of error (5%) and n = sample size.

n = (Z α/2)2*p (1 − p)/d2 n = (1.96)2*0.305*0.695/(0.05)2, adding 10% for the incomplete chart, we got 359.

For the second objective, the sample size was determined by double proportion formula using Epi Info version 7.2 considering different predictors^17^ (Table 1).

**Table 1:** Sample size determination to determine the predictor of attrition among HIV-infected children on ART in Amhara Comprehensive Specialized Hospital, Northwest Ethiopia, 2022.

Assumption	Variables			
	CPT prophylaxis	Baseline WHO staging	Baseline Hgb	Weight for age
CI	95%	95%	95%	95%
Power	80%	80%	80%	80%
Ratio (r)	1	1	1	1
HR	2.5	2.3	2.7	3.1
P1	67.4	55.8	76.7	41.8
P2	25.3	20.9	23.9	15.5
Sample size	58	77	38	117

P1: is the percent of exposed with the outcome, P2: is the percent of non-exposed with the outcome, Zα/2: is taking CI 95%, Zβ: 80% power and r is the ratio of non-exposed to exposed 1:1. Then the largest sample size was 359, so we considered this as the final sample.

### Sampling techniques and procedures

Initially, lottery methods were used to select those four hospitals out of eight. The registration numbers of under-15 years old children started ART from June 14, 2014, to June 14, 2022, were collected from the databases of each selected hospital. After that, the sample size was proportionally allocated to each hospital based on the number of children started ART as follows: 127 out of 241 from University of Gondar hospital, 111 out of 212 from Felege Hiwot hospital, 70 out of 133 from Debre Markos hospital, and 51 out of 98 from Debre Tabor hospital. A total of 359 medical charts were reviewed, of which 15 charts were excluded based on the criteria. Finally, a computer-generated simple random sampling method was used.

### Study variables

The dependent variable was the attrition status. The independent variables include socio-demographic characteristics of the child and caregivers (age, sex, residence, parental live status, HIV status, educational status, relation with the child, and marital status). Baseline clinical and laboratory related characteristics (WHO clinical stage, CD4 count/%, hemoglobin, developmental status, nutritional status, TB/HIV co-infection, and presence of OI) and ART and other treatment-related predictors (types of regimen, ART adherence, drug side effects, and OI prophylaxis).

### Operational definitions

Attrition (i.e., an event) is defined as a child either dead or lost to follow-up (LTFU) reported in the child’s medical record during the follow-up period^8^.

**Lost to follow up:** If children did not return to the ART visit within 90 days or more after their last scheduled appointment^6^.

**Mortality:** Recorded as “dead” on the child’s medical card.

**Censored:** Censors are children who were transferred out and those who were active on ART.

**WHO clinical stage:** It is a classification of HIV patients at ART initiation based on the clinical illnesses as defined by WHO clinical staging, and in this study it was categorized as non-advanced HIV disease (WHO stage I&II) and advanced HIV disease (WHO stage III&IV)^18^.

**Incomplete records:** Those records lacking information on the date of ART initiation and unknown or unrecorded outcomes^19^.

### Data collection tool and procedures

The data abstraction tool was adapted from the Ethiopian Federal Ministry of Health HIV/AIDS care and treatment follow-up forms. The most current laboratory test results before initiation of ART were used as a baseline, but if there were no pre-ART test results registered, values obtained within 1 month of initiation were used as a baseline. The data were collected from August 11 to September 11, 2022, by four trained BSc nurses using Kobo Toolbox software. Two MSc nurses who had an ART mentoring certificate were selected for supervision. The charts were retrieved from the computer database using medical registration numbers by a recruited diploma HIT data clerk.

### Data quality control

A pretest was conducted on ten patient charts at Debre Tabor Comprehensive Specialized Hospital. After that, the necessary modifications were made. Data quality was also maintained by proper recruitment and one-day training for both the data collectors and supervisors separately. Both the principal investigator and supervisors closely supervised the data collection process, completeness, and consistency of the data onsite and/or on the server daily. All four data collectors have basic and comprehensive HIV care and treatment training. Additionally, one data collector has also experienced with mobile data collection. The two supervisors have ART mentoring certificates and an electronic data collection certificate as team leaders.

### Data processing and analysis

The data were checked for completeness and consistency, and exported to STATA version 14 for analysis. The WHO anthro and anthro plus were used to produce Z scores to define the nutritional status of children. The missing data was handled via multiple imputations.

We conducted an exploratory analysis to assess the normality of the data and identify any outliers. Categorical data were described using frequency tables and percentages. We generated a Kaplan–Meier curve to estimate the median time to attrition over the follow-up period and used log-rank tests to compare survival curves across various categories of predictor variables. Additionally, a life table was constructed to estimate the cumulative probability of attrition at different time intervals. To provide a standardized measure, the incidence rate of attrition was calculated and presented as per 1000 child-months of observation. We conducted a univariate analysis using Cox proportional hazard regression to examine the relationship between attrition and each independent variable. Variables with a p-value of 0.25 or less were considered for inclusion in the multivariable analysis to identify independent predictors of attrition. To assess multicollinearity, we checked the variance inflation factor (VIF), and the average VIF was found to be 1.25, indicating no significant multicollinearity.

Additionally, we assessed the proportional hazard assumptions by conducting the Schoenfeld residual test (p > χ^2^ = 0.5322). Furthermore, we calculated Harrell's C (C = 0.8837), which suggests that this study can accurately predict the ordering of survival times for pairs of children 88.4% of the time based on the observed variables included in the model. To evaluate the fitness of the Cox regression model to the data, we examined the Cox–Snell residuals. The Nelson–Aalen hazard function closely followed the 450 line. In general, we can conclude that the model fits the data successfully.

In the final Cox proportional hazard model, statistical significance was determined at a p-value of less than 0.05. The presence and magnitude of associations were summarized using adjusted hazard ratios (AHR) with corresponding 95% confidence intervals (CIs). The study findings were presented through texts, tables, and graphs.

### Ethics approval and consent to participate

Ethical clearance was obtained from the school of nursing on behalf of the University of Gondar institutional ethical review board dated May 6, 2022 with a reference number of SNEC 222/2022 and a consent waiver was obtained from the school of Nursing Ethical Committee, University of Gondar. Then, the data were collected after obtained a permission letter from each hospital and the Amhara public health institute. The data abstraction tool's information was kept private. This study was followed the principles of Helsinki Declaration.

## Results

### Socio-demographic characteristics of the child and child’s caregiver information

A total of 359 medical records of children on ART were retrieved and reviewed. Of these, 344 charts were included in the final analysis. More than half (54.6%) of the children were female, and more than two-thirds of them (69.5%) were from urban areas. The median age of the child at the time of ART initiation was 5 years, with an IQR of 2.25–10. More than one fifth (21.2%) of children whose parental status was reactive develop attrition (Table 2).

**Table 2:** Socio-demographic characteristics of children who are on ART at Amhara Comprehensive Specialized Hospital, from 2014–2022.

Variables	Categories	Attrition n (%) N = 90	Censored n (%) N = 254	Total n (%) N = 344
Sex	Male	34 (9.9)	122 (35.5)	156 (45.4)
	Female	56 (16.2)	132 (38.4)	188 (54.6)
Child’s age	< 5 years	46 (13.4)	110 (32)	156 (45.4)
	5–9 years	32 (9.3)	62 (18)	94 (27.3)
	10–14 years	12 (3.5)	82 (23.8)	94 (27.3)
Residence	Urban	56 (16.3)	183 (53.2)	239 (69.5)
	Rural	34 (9.9)	71 (20.6)	105 (30.5)
Educational status of caregiver	No education	22 (6.4)	34 (9.9)	56 (16.3)
	Primary	39 (11.3)	99 (28.8)	138 (40.1)
	Secondary	14 (4)	89 (25.9)	103 (29.9)
	College and above	15 (4.4)	32 (9.3)	47 (13.7)
Marital status	Single	18 (5.2)	38 (11.1)	56 (16.3)
	Married	56 (16.3)	174 (50.6)	230 (66.9)
	Divorced	8 (2.3)	31 (9)	39 (11.3)
	Widowed	8 (2.3)	11 (3.2)	19 (5.5)
Parental status	Both alive	56 (16.3)	196 (57)	252 (73.3)
	One/both deceased	34 (9.9)	58 (16.9)	92 (26.7)
Relation of caregiver	Parent	63 (18.3)	220 (64)	283 (82.3)
	Non-parent	27 (7.9)	34 (9.9)	61 (17.7)
HIV status of caregiver	Reactive	73 (21.2)	218 (63.4)	291 (84.6)
	Non-reactive	17 (4.9)	36 (10.5)	53 (15.4)

### Baseline clinical and laboratory related information

Baseline clinical and laboratory related characteristics of the study participants were summarized (Table 3).

**Table 3:** Baseline clinical and laboratory related information of children on ART in Amhara region comprehensive specialized hospitals, Northwest Ethiopia, 2022.

Variables	Categories	Attrition n (%) N = 90	Censored n (%) N = 254	Total n (%) N = 344
WHO staging	Stage I and II	45 (13.1)	191 (55.5)	236 (68.6)
	Stage III and IV	45 (13.1)	63 (18.3)	108 (31.4)
CD4 count or percent	Below the threshold	29 (8.4)	60 (17.5)	89 (25.9)
	Above the threshold	61 (17.7)	194 (56.4)	255 (74.1)
Functional/developmental status	Working/appropriate	48 (14)	187 (54.4)	235 (68.4)
	Ambulatory/delayed	33 (9.6)	61 (17.3)	94 (26.9)
	Bedridden/regressive	9 (2.6)	6 (1.7)	15 (4.4)
Hemoglobin	< 10 mg/dl	25 (7.3)	11 (3.2)	36 (10.5)
	> = 10 mg/dl	65 (18.9)	243 (70.6)	308 (89.5)
Opportunistic infection	Yes	28 (8.1)	53 (15.4)	81 (23.5)
	No	62 (18.1)	201 (58.4)	263 (76.5)
TB-HIV co-infection	Yes	27 (7.9)	31 (9)	58 (16.9)
	No	63 (18.3)	223 (64.8)	286 (83.1)
Underweight	Normal	73 (21.2)	195 (56.7)	268 (27.9)
	Underweight	17 (5)	59 (17.1)	76 (22.1)
Wasting	Normal	75 (21.8)	193 (56.1)	268 (77.9)
	Wasting	15 (4.4)	61 (17.7)	76 (22.1)
Stunting	Normal	64 (18.6)	172 (50)	236 (68.6)
	Stunting	26 (7.6)	82 (23.8)	108 (31.4)

### ART and other treatment related information

Nearly half of the ART drug combinations (49.7%) were ABC-based. Nearly one fourth (22.4%) of children who were on CPT develop attrition. Moreover, about three-fourths (74.4%) of children had optimal ART adherence (Table 4).

**Table 4:** Treatment-related information of children on ART at Amhara region comprehensive specialized hospitals, Northwest Ethiopia, 2022.

Variables	Categories	Attrition n (%) N = 90	Censored n (%) N = 254	Total n (%) N = 344
Initial ART based regimens	ABC based	50 (14.5)	121 (35.2)	171 (49.7)
	AZT based	26 (7.6)	104 (30.2)	130 (37.8)
	TDF based	10 (2.9)	24 (7)	34 (9.9)
	D4T based	4 (1.2)	5 (1.4)	9 (2.6)
Drug side effect	Yes	47 (13.7)	10 (2.9)	57 (16.6)
	No	43 (12.5)	244 (70.9)	287 (83.4)
CPT use	Yes	77 (22.4)	216 (62.8)	293 (85.2)
	No	13 (3.8)	38 (11)	51 (14.8)
IPT use	Yes	44 (12.8)	158 (45.9)	202 (58.7)
	No	46 (13.4)	96 (27.9)	142 (41.3)
ART adherence	Optimal	56 (16.3)	200 (58.1)	256 (74.4)
	Suboptimal	34 (9.9)	54 (15.7)	88 (25.6)

### Incidence and time to develop attrition after initiation of ART

The study participants were followed for a minimum of 0.8 months and a maximum of 90.1 months. During the follow-up, 90 (26.2%) experienced attrition with death, and the losses to follow-up were 30 (8.7%) and 60 (17.4%), respectively. The total person-months of the cohort were 11,067.97 child-months or 922.33 child-years of observation. The overall incidence rate of attrition was found to be 8.13 (95% CI 6.6, 10) per 1000 child-months or 9.8 (95% CI 7.9, 11.9) per 100 person years of observations.

**Figure 1:**
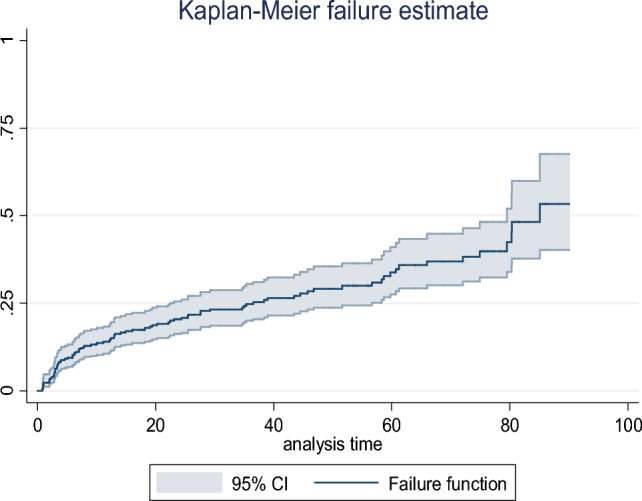
The overall Kaplan–Meier failure function with 95% CI of children on ART in Amhara region comprehensive specialized hospitals, Northwest Ethiopia, 2022.

Regarding the timing of attrition, 44 (48.9%) and 46 (51.1%) patients experienced attrition within the first year and after 1 year of ART initiation, respectively. The median survival time was 85.1 months, and the Kaplan–Meier estimation showed that the cumulative probability of attrition at 6, 12, and 24 months and the end of follow-up after the initiation of ART were 0.1025, 0.1410, 0.2054, and 0.5723, respectively (Fig. 1).

### Survival function of different predictor variables

**Figure 2:**
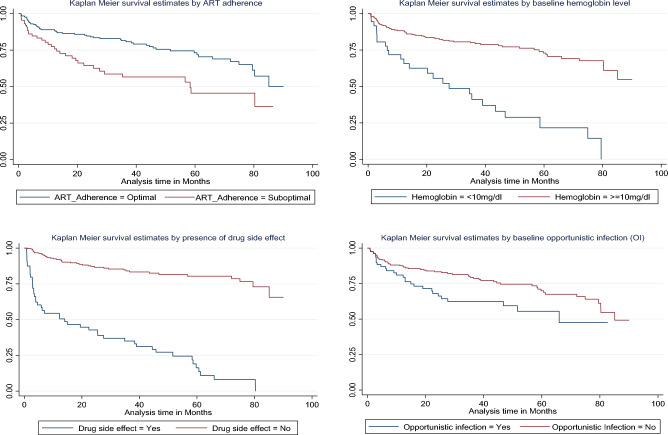
Kaplan–Meier of attrition-free survival of four main predictor variables among children on ART in Amhara Comprehensive Specialized Hospitals, Ethiopia, 2022.

To test the equality of the survival curves of different categorical predictors of attrition, the Kaplan–Meier and Cochran-Mantel Haenszel Log rank tests were employed and the Kaplan–Meier attrition-free survival probability of the main predictor variable were estimated (Fig. 2).

### The goodness of fit of the proportional hazard model

**Figure 3:**
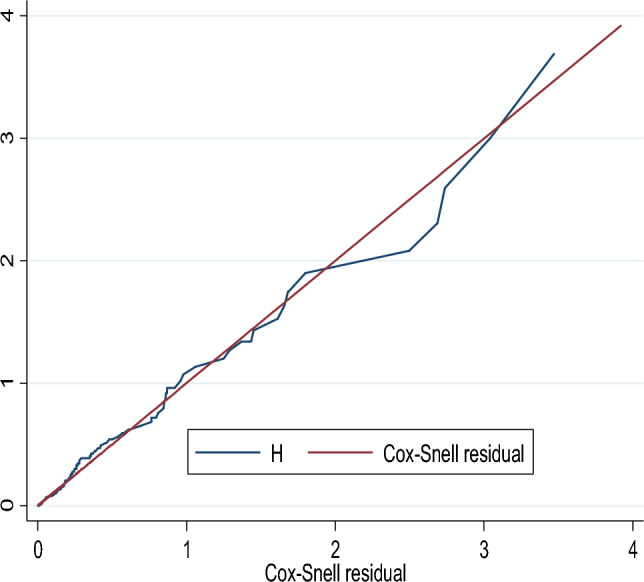
Graph of Nelsen-Aalen cumulative hazard function against Cox–Snell residuals.

The proportional model assumption was checked by using the Schoenfeld residuals test together with the graphical test and it was found that a global p-value of 0.5322. Besides, the overall goodness of model fitness was checked through the Cox–Snell residual (Fig. 3). Based on that finding, we would conclude that the final model fits the data very well.

### Predictors of attrition among HIV-infected children on ART

In the bivariable Cox regression analysis, fourteen variables were predictors of attrition with a p-value of < 0.25. However, in multivariable analysis, baseline hemoglobin, ART adherence, parental status, presence of baseline OI, and drug side effects were identified as independent predictors of attrition at the 5% significance level.

Children with baseline hemoglobin < 10 mg/dl had a nearly four-fold increased hazard of attrition than children with hemoglobin levels > = 10 mg/dl (AHR 3.94; 95% CI 2.32, 6.7). Likewise, those children who had suboptimal adherence had a nearly two-fold increased the hazard of attrition than children who had optimal adherence (AHR 1.96, 95% CI 1.23, 3.13). In addition, children with baseline OI increase the risk of attrition by 1.8 times (AHR 1.8, 95% CI 1.17–2.96) compared to those who do not have baseline OI. Moreover, children who had experienced drug side effects surge the hazard by eight times compared to those children who did not experience drug side effects (AHR 8.3; 95% CI 4.93, 13.84) (Table 5).

**Table 5:** Bivariable and multivariable cox regression for predictors of attrition among children.

Variables	Categories	Attrition (%) N = 90	Censored (%) N = 254	CHR (95% CI)	AHR (95% CI)	p-value
Sex	Male	34 (9.9)	122 (35.5)	1	1	
	Female	56 (16.2)	132 (38.4)	1.34 (0.88, 2.06)	0.73 (0.44, 1.19)	0.212
Child’s age (years)	< 5	46 (13.4)	110 (32)	2.0 (1.06, 3.79)	0.99 (0.50, 1.95)	0.977
	5–9	32 (9.3)	62 (18)	1.84 (0.94,3.61)	0.72 (0.34, 1.52)	0.386
	10–14	12 (3.5)	82 (23.8)	1	1	
Residence	Urban	56 (16.3)	183 (53.2)	1	1	
	Rural	34 (9.9)	71 (20.6)	1.61 (1.05, 2.47)	0.81 (0.49, 1.35)	0.421
Relation of caregiver	Parent	63 (18.3)	220 (64)	1	1	
	Non-parent	27 (7.9)	34 (9.9)	2.29 (1.46, 3.61)	1.51(0.89, 2.57)	0.127
WHO staging	Stage I and II	45 (13.1)	191 (55.5)	1	1	
	Stage III and IV	45 (13.1)	63 (18.3)	2.6 (1.72, 3.93)	0.84 (0.48, 1.47)	0.539
CD4 count or percent	Below the threshold	29 (8.4)	60 (17.5)	1	1	
	Above the threshold	61 (17.7)	194 (56.4)	0.58 (0.37, 0.91)	0.83 (0.46, 1.5)	0.539
Hemoglobin	< 10 mg/dl	25 (7.3)	11 (3.2)	4.1 (2.57, 6.53)	3.94 (2.32, 6.7)	0.001*
	> = 10 mg/dl	65 (18.9)	243 (70.6)	1	1	
Opportunistic infection	Yes	28 (8.1)	53 (15.4)	1.79 (1.14, 2.82)	1.8 (1.17, 2.96)	0.02*
	No	62 (18.1)	201 (58.4)	1	1	
TB-HIV co-infection	Yes	27 (7.9)	31 (9)	2.75 (1.75, 4.32)	1.38 (0.82, 2.35)	0.228
	No	63 (18.3)	223 (64.8)	1	1	
Drug side effect	Yes	47 (13.7)	10 (2.9)	8.32 (5.5, 12.64)	8.3 (4.93, 13.84)	0.001*
	No	43 (12.5)	244 (70.9)	1	1	
IPT use	Yes	44 (12.8)	158 (45.9)	1	1	
	No	46 (13.4)	96 (27.9)	1.7 (1.12, 2.58)	1.6 (1.0, 2.64)	0.05
ART adherence	Optimal	56 (16.3)	200 (58.1)	1	1	
	Suboptimal	34 (9.9)	54 (15.7)	2.12 (1.38, 3.25)	1.96 (1.23, 3.13)	0.005*

*CHR* crude hazard ratio, *AHR* adjusted hazard ratio, *ART* antiretroviral therapy, *CD4* cluster of differentiation 4, *IPT* isoniazid prophylactic therapy, *OI* opportunistic infection, *TB* tuberculosis, *WHO* World Health Organization.

*P value < 0.05.

## Discussion

The objective of this study was to examine the occurrence and factors associated with attrition among children infected with HIV who were receiving antiretroviral therapy (ART). The findings of this study revealed that the overall incidence rate of attrition was 9.8 (95% CI 7.9, 11.9) per 100 person-years of observation (PYO). Factors such as lower baseline hemoglobin levels, suboptimal adherence to treatment, presence of opportunistic infections (OI) at baseline, and experiencing drug side effects were identified as predictors of attrition.

The current study was in congruent with the previous studies conducted in Ethiopia^8^ and Côte d’Ivoire^20^, in which the attrition rate was 8.3 and 9.75 per 100 PYO, respectively. The possible explanation for this similarity could be attributed to the researcher's use of a consistent operational definition, as well as the similarity in socio-demographic characteristics among the study participants. Additionally, the adherence to WHO and Ministry of Health standards for monitoring and recording data during the follow-up period may have contributed to the resemblance. Furthermore, the similarity in the level of awareness regarding healthcare-seeking behavior among these populations could have played a role^21^.

However, the present study was higher than the studies conducted in Myanmar and Ethiopia^2,17^, in which the attrition rate was 4 and 6.6 per 100 PYO, respectively. This variation might be due to socio-demographic characteristics, the study and the follow-up period difference because most deaths and LTFU occurred early after ART initiation. Moreover, this dissimilarity might be due to the access and quality of healthcare services and healthcare-seeking behavior variation among those populations and countries.

On the contrary, the present study was lower than studies conducted in Southern Ethiopia^21^, West Africa^11^, Uganda^22^, Sub-Saharan Africa^23^, and East African countries^24^. Possible explanations for this phenomenon may include advancements in healthcare services and the availability of improved and more tolerable antiretroviral therapy (ART) medications. Additionally, changes in guidelines, such as increased frequency of visits with enhanced adherence support and greater involvement of families in the care process, could have contributed to the observed differences. Another potential factor could be the variation in sample sizes, as previous studies conducted in East Africa and West Africa may have involved larger cohorts.

The current study also revealed that children who had low hemoglobin levels (< 10 mg/dl) were 3.94 times more at risk of attrition than their counterparts (> = 10 mg/dl). This finding was in agreement with other studies^8,17,21^. This might be anemia, which is associated with reduced immune defense mechanism, leading in reduced tolerance and alter the pharmacokinetics effect of ART drugs, which leads to exhaustion and non-compliance with ART follow-up, resulting in LTFU and death. In addition, it is worth noting that certain ART regimens have been linked to the development of anemia, which could worsen pre-existing conditions. Another possible explanation is that decreased ART tolerance may occur due to reduced absorption and the impact of immune defense mechanisms affected by anemia. To support this conclusion, it is important to highlight that approximately two-thirds (37.8%) of the participants in our study initially received a zidovudine-containing regimen. Among those, 7.6% of the children experienced attrition during the follow-up period.

Furthermore, children who had suboptimal adherence to ART drugs at baseline were 1.96 times more likely to experience attrition compared with those who had optimal adherence. This finding is similar with the previous studies^21^. This might be suboptimal adherence, which adds to increased viral replication, ART drug resistance and poor patient outcomes. As a result, children living with HIV/AIDS may become discouraged, too shattered, hopeless, and fatigued in adhering to their ART visits. Additionally, children rely on others to attend their follow-up appointments and ensure timely and proper medication administration. Hence, issues related to caretakers may also influence adherence. Therefore, it is essential to frequently visit the health facility and increase the level of adherence through integrated service approach to minimize attrition and increasing retention of cares.

Similarly, children who had experienced adverse drug effects had an eight-fold increased risk of attrition compared with those who did not have adverse drug effects. This finding was congruent with other studies^25–27^. This occurrence could be attributed to the presence of numerous burdens and intolerances associated with the medication administered to patients. These burdens and intolerances have the potential to exacerbate the progression of the disease, hinder the effectiveness of the treatment, and ultimately contribute to the loss of patient follow-up and fatal outcomes. Therefore, stakeholders should consider implementing vigilant monitoring, creating personalized treatment plans, providing comprehensive support for medication adherence, and fostering collaborative decision-making.

Moreover, children who had baseline OI increased the hazard of attrition compared with children who had no baseline OIs. This findings was similar with other findings that have been revealed as OI is a potential predictor of attrition^26^. This situation could be attributed to the presence of various illnesses that impose multiple burdens on individuals. These illnesses may have detrimental effects on the psychological well-being of patients and weaken their immune system. As a consequence, the patients may experience challenges in adhering to their treatment, leading to attrition and potentially resulting in fatal outcomes. Hence, it is advisable for stakeholders to prioritize early identification and treatment, deliver comprehensive healthcare services, provide education and counseling, and offer supportive care, especially to children who have a baseline OI.

## Limitation and strength of the study

This study accounted censored observations for analysis, was multicenter, and used a longer follow-up period for better estimate the incidence of attrition, but the source of data was secondary chart review, so some vital variables such as viral load were left unevaluated and those excluded charts with missing data may underestimate or overestimate the findings.

## Conclusion and implication of the study

The incidence rate of attrition was found to be relatively high. Decreased hemoglobin, drug side effects, baseline OI, and suboptimal adherence were independent predictors of attrition. Therefore, it is crucial to detect and give special emphasis to those identified predictors promptly.

The findings of this study have important implications for healthcare providers, policymakers, and researchers in the field of pediatric HIV care. To disseminate these findings, we plan to present our results at national and international conferences focused on HIV/AIDS, pediatric medicine, and public health. By sharing our findings with fellow researchers and healthcare professionals, we aim to contribute to the collective understanding of factors influencing attrition rates among children on ART and facilitate the identification of strategies to improve retention in care. Besides, by collaborating with these stakeholders, we can facilitate the integration of our findings into national and international guidelines for pediatric HIV care, thus promoting quality improvement initiatives and interventions to reduce attrition rates among children on ART.

Moreover, by disseminating our findings widely and engaging with diverse stakeholders, we aim to raise awareness about the challenges associated with attrition among children on ART and contribute to the development of comprehensive and sustainable interventions that can enhance the long-term health outcomes of this vulnerable population.

## Data Availability

The datasets of the current study will be available upon reasonable request from the corresponding author and share it in the supplementary file. You can get the data set from the corresponding author up on resonable request via alexb7298@gmail.com

## Abbreviations

AHR: Adjusted hazard ratio
AIDS: Acquired immune deficiency syndrome
ART: Antiretroviral therapy
HIV: Human immunodeficiency virus
OI: Opportunistic infection
TB: Tuberculosis
WHO: World Health Organization
